# Immunohistochemical study of pituitary cells in wild and captive *Salminus hilarii* (*Characiformes*: *Characidae*) females during the annual reproductive cycle

**DOI:** 10.1186/2193-1801-2-460

**Published:** 2013-09-13

**Authors:** Renato Massaaki Honji, Rafael Henrique Nóbrega, Matias Pandolfi, Akio Shimizu, Maria Inês Borella, Renata Guimarães Moreira

**Affiliations:** Departamento de Fisiologia, Instituto de Biociências, Universidade de São Paulo, Rua do Matão, Trav. 14, N° 321, 05508-090 São Paulo, SP Brazil; Instituto de Ciências Biomédicas, Universidade de São Paulo, Av. Prof. Lineu Prestes, N° 1524, 05508-900 São Paulo, SP Brazil; Departamento de Biodiversidad y Biología Experimental, Facultad de Ciencias Exactas y Naturales, Universidad de Buenos Aires, Ciudad Universitaria (C1428EHA), Buenos Aires, BA Argentina; National Research Institute of Fisheries Sciences, Fisheries Research Agency, 236-8648 Kanazawa, Yokohama, Japan

**Keywords:** Gonadotropins, Prolactin hormone, Somatolactin hormone, Growth hormone, Reproductive dysfunction

## Abstract

Freshwater fish that live exclusively in rivers are at particular risk from fragmentation of the aquatic system, mainly the species that migrate upriver for reproduction. That is the case of *Salminus hilarii*, an important migratory species currently classified as “almost threatened” in the São Paulo State (Brazil), facing water pollution, dam construction, riparian habitat destruction and environmental changes that are even more serious in this State. Additionally, this species show ovulation dysfunction in captivity. Our studies focused on the identification and distribution of the pituitary cell types in the adenohypophysis of *S. hilarii* females, including a morphometric analysis that compares pituitary cells from wild and captive broodstocks during the reproductive annual cycle. The morphology of adenohypophysial cells showed differences following the reproductive cycle and the environment. In general, optical density suggested a higher cellular activity during the previtellogenic (growth hormone) and vitellogenic (somatolactin) stages in both environments. Additionally, the nucleus/cell ratio analysis suggested that growth hormone and somatolactin cells were larger in wild than in captive females in most reproductive stages of the annual cycle. In contrast, prolactin hormone showed no variation throughout the reproductive cycle (in both environments). Morphometrical analyses related to reproduction of *S. hilarii* in different environmental conditions, suggest that somatolactin and growth hormone play an important role in reproduction in teleost and can be responsible for the regulation of associated processes that indirectly affect reproductive status.

## Introduction

*Salminus hilarii* (Valenciennes, 1850), commonly named tabarana, is one important migratory freshwater fish of the Brazilian ichthyofauna. It is a potamodromous (rheophilic) and carnivorous fish species occupying the top of the food chain and has a geographical distribution from the upper Paraná River Basin to São Francisco, Tocantins, Alto Amazonas, and Alto Orinoco Basins (Mirande 2010). In *S. hilarii*, like other migratory fish species (*Brycon orbignyanus*, *S. brasiliensis*, *Pseudoplatystoma corruscans*), the upstream migration is necessary to complete the development of their gonads and reproduce (Agostinho et al. 2003; Andrade et al. 2006), considering that the spawning behavior in *S. hilarii* takes place in the upper region of minor tributaries in this Basin. When migration is not possible, tabarana females do not ovulate, and consequently do not spawn (Honji et al. 2009,2011). Additionally, *S. hilarii* in the region of the upper reaches of the Tietê River (the river included in Paraná River Basin) has a great ecological importance given its high degree of environmental selectivity, it can be used as an accurate environmental indicator, considering the limited occurrence of this species just in a few unpolluted regions of the Paraná River Basin (Lima-Junior 2003; Villares-Junior et al. 2007). Moreover, this species is currently classified as “almost threatened” in the São Paulo State of Brazil (São Paulo 2008). The endangered status of this species deserves special attention and urgent action addressing knowledge of its reproductive physiology, which is the basic premise for the program of fish restocking in the Tietê River (Honji et al. 2011).

Changes in the natural environment due to threats, such as pollution (domestic, industrial, and agricultural pollution), and alteration or obstruction of river flows (like dam’s construction) are severe in the upper reaches of the Tietê River (Silva et al. 2006), resulting in a serious impact on the ichthyofauna, mainly in rheophilic fish in South America. In addition, overfishing is evident in some parts of the Paraná Basin, and obstructions in rivers (i.e., construction of dams) affect the flow of many waterways in South America, which are often so deeply dammed that they sometimes look like a chain of reservoirs (Silva et al. 2006).

Some studies have focused on the effects of environmental changes on the endocrine system. The environmental factors considered include fluctuations in salinity and variations in temperature and photoperiod, which induce internal adjustments within the fish (Vargas-Chacoff et al. 2009; Onuma et al. 2010). Changes in reproductive status also lead to adjustments, such as an altered hormone profile during the annual reproductive cycle (Mousa and Mousa 2000; Onuma et al. 2010). Other studies have focused on animals in differing conditions, such as wild and cultured broodstocks, with the objective of identifying possible endocrine failure, which might be related to the reproductive dysfunction observed in fish farmed broodstocks (Guzmán et al. 2009).

Additionally, as described by Lubzens et al. (2010), in recent years, due to the decline in natural resources due to overfishing and a growing demand for the diversification of marketable products, there is an urgent need for new aquaculture species. However, many commercial fish farms depend on the collection of wild broodstock from the natural environment and their transfer to captivity for artificial reproduction. Unfortunately, the maintenance of broodstock in captivity is not completely successful because many fish exhibit reproductive dysfunctions when reared in captivity (Mylonas et al. 2010). The reproductive dysfunction in captive conditions is the common problem faced in the aquaculture and/or conservation aquaculture of neotropical migratory fish, including *S. hilarii*. Therefore studies of neotropical migratory fish in both wild and captive environments have relevance.

These facts emphasize the importance of the study of neotropical migratory fish in both wild and captive environments. Nevertheless, there are few studies that aim to characterize the reproductive cycle (Andrade et al. 2006; Honji et al. 2009) and offspring (Araújo et al. 2012) of *S. hilarii*, and only preliminary data on the endocrine features of this important potamodromous species (Amaral et al. 2007). Thus, more information regarding the reproductive physiology of *S. hilarii* during gonadal maturation in both wild and captive broodstocks is needed. Additionally, any new insights into the neural and hormonal processes that control the reproductive activities can be applied in conservation programs.

Taking these points into consideration, our studies have focused on identifying and localizing the different pituitary cell types in the adenohypophysis of *S. hilarii* female, including a morphometric analysis that compares the pituitary cells of females from wild with captive females during the previtellogenic, vitellogenic and regression phases of the reproductive annual cycle. These topics can be used as the basis for future studies in this species, since *S. hilarii* females present an endocrine dysfunction when transferred from wild environment to captivity.

## Material and methods

### Fish collection

**Figure 1:**
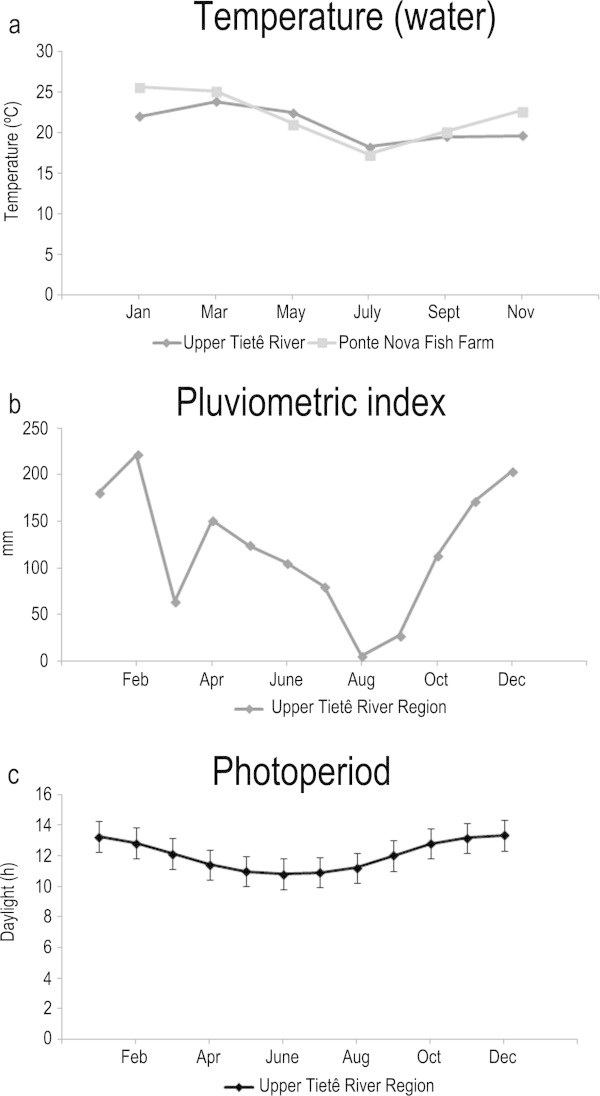
**Environmental data obtained from the Brazilian Government Agencies. a)** Water temperature (Upper Tietê River and Ponte Nova Fish Farm); **b)** pluviometric index; **c)** Photoperiod. Sources: “Departamento de Água e Energia Elétrica (DAEE)”, “Instituto Agronômico e Geofísico (IAG-USP), and “Companhia de Tecnologia de Saneamento Ambiental (CETESB)”.

Sexually adult *S. hilarii* females were captured from April 2004 to August 2006 by artisanal fisheries in the region of the “Alto Tietê” Basin (Honji et al. 2009). For the wild group (26 animals sampled, Table 1), fish were caught at two locations in the Tietê River, between Mogi das Cruzes and Biritiba Mirim, São Paulo State, Brazil. At the first location, the fish were caught in lotic waters in the main channel of the Tietê River (23° 32’ 45.3”S and 46° 08’ 03.2” W), the same characteristic of the second point, that was also in the Tietê River (23° 34’ 36.5”S e 45° 54’ 23.9” W), but just downstream the Ponte Nova Dam. Initially, we considered that fish sampled in points 1 and 2 could have a different pattern of ovary development, but according to previous analyses (Honji et al. 2009) animals from both points at Tietê River were similar, therefore they were considered as one group. Temperature, photoperiod, and pluviometric index (Figure 1) were obtained from the Brazilian Government Agencies “Departamento de Água e Energia Elétrica (DAEE)”, “Instituto Agronômico e Geofísico (IAG-USP), and “Companhia de Tecnologia de Saneamento Ambiental (CETESB)”, which monitored these data in the Tietê River Basin. More details of the sampled animals were described by Honji et al. (2009).

**Table 1:** **Biometrical parameters in different environments, ovarian maturation stages and gonadosomatic index (GSI)**

Environment	Maturation stage	N	Total length (cm)	Total weight (g)	Gonads weight (g)	GSI (%)
Wild	Previtellogenic	11	38.33 ± 3.47	574.33 ± 144.49	4.34 ± 1.33^a^	0.76 ± 0.09^a^
	Vitellogenic	11	42.37 ± 1.17^a*^	945.17 ± 94.97	110.43 ± 21.96^b^	11.41 ± 1.58^b^
	Regression	4	47.50 ± 5.00	975.55 ± 153.25	30.89 ± 14.07^ab^	2.24 ± 0.22^a*^
Captivity	Previtellogenic	10	35.75 ± 0.66	519.50 ± 56.58	6.59 ± 1.83^a^	1.24 ± 0.31^a^
	Vitellogenic	6	35.33 ± 2.32^**^	593.33 ± 107.24	66.93 ± 10.40^b^	11.52 ± 1.46^b^
	Regression	6	36.85 ± 0.85	648.00 ± 44.00	78.00 ± 15.00^b^	12.25 ± 3.15^b**^

Values followed by different letters (a,b) are significantly different during the reproductive cycle. Values followed by different symbols (*) are significantly different between the environments (*P < 0.05*).

The captive group (22 animals collected, Table 1) was composed by animals previously sampled at Tietê River (in 2000) and maintained at the Ponte Nova Fish Farm (23° 35’ 33.8”S and 45° 58’ 09.1” W), Salesópolis, São Paulo State, Brazil, for five years in captivity (a period during which they never spawned). During the experimental period (2005–2006), the animals of this group were randomly distributed in two ponds at the Ponte Nova Fish Farm and fed with commercial extruded feed for carnivorous fish (40% crude protein) at a feeding rate of 2% of the biomass/day, offered to the animals in 2 daily portions. Water temperature and dissolved oxygen were monitored daily with an oximeter (YSI model 55).

*S. hilarii* sampled in the river and the ones in captivity were transported to the laboratory of the fish farm (the distance of the sampling point to the laboratory was about 15 km for first point) is plastic bags, with water and oxygen. To minimize the stress of capture, the animals sampled in the river were maintained for 24 hours in running water in the fish farm. Fish were anesthetized with tricaine methanesulfonate (MS-222; Sigma Diagnostics INS, St. Louis, MO, USA; 1 g MS-222: 10 l water), neutralized with sodium bicarbonate (1 g: 10 l water) and killed by decapitation (according to the institutional animal care protocols (Comissão de Ética em Manipulação e Experimentação Animal – CEMEA: approval 0.28.04 – 21/05/2004). Total and standard length (in centimeters) and total body weight (in grams) were registered for each animal.

The pattern of oocyte development in wild and captive *S. hilarii* was previously described (Honji et al. 2009) and the sampling were herein chosen to reflect the reproductive stages observed in wild females, as previously determined (Honji et al. 2009), and adjusted herein in 3 stages: previtellogenic (April-August), which comprises the animals before the beginning of exogenous vitellogenesis (perinucleolar and cortical-alveolar oocytes), vitellogenic (September-December), which encompass the vitellogenic female (vitellogenic oocytes), and regression (January-March), which include the animals that shows atretic oocytes. Additionally, the gonadosomatic index (GSI) was calculated (Vazzoler 1996).

### Histological analysis of the pituitary gland

The infundibular recess was very thin, and at the time of extracting the pituitary gland, it was broken and could not be extracted together with the brain. The pituitary gland was dissected without the brain, fixed in Bouin’s solution for 24 hours and dehydrated through a series of increasing ethanol dilutions. Glands were then cleared in dimethylbenzene solutions and embedded in Paraplast® according to normal histological procedures (Behmer et al. 1976). Serial sections (5 μm thick) were obtained from most specimens and stained with Mallory trichrome and periodic acid-Schiff (PAS). Sections were then examined and documented using a computerized image analyzer (LEICA DM 1000 light microscope, LEICA DFC295 photographic camera and image capture LEICA Application Suite Professional, LAS V3.6).

### Immunohistochemical analysis of the pituitary gland

For immunohistochemical analysis, pituitary tissue sections were immunostained according to SABC complex (streptavidin-biotin-peroxidase complex, DakoCytomation LSAB_2_® System HRP Liquid DAB – Ref. 0673). Briefly, sections were deparaffinized in xylene (dimethylbenzene), rehydrated in decreasing ethanol dilutions, washed in phosphate-buffered saline (PBS, pH 7.4), treated with 0.3% H_2_O_2_ in PBS for 15 min at room temperature to inactivate endogenous peroxidase activity, washed in PBS again and treated with 5% non-fat dry milk in PBS for 30 min at room temperature to inactivate non-specific sites (blocking solution). Next, the sections were incubated with the specific primary antiserum overnight at 4°C (the antiserum and dilutions used are detailed in Table 2). After washing with PBS, the samples were treated with biotinylated secondary antibody (Universal DakoCytomation LSAB + System-HRP, peroxidase), washed again with PBS, covered with the SABC complex and visualized with 3,3’-diaminobenzidine in a chromogen solution and DAB substrate buffer (imidazole-HCl buffer, pH 7,5, containing hydrogen peroxide and an anti-microbial agent; DAB – DakoCytomation LSAB_2_® System HRP). Samples were counterstained with hematoxylin and mounted with Erv-Mount (Erviegas Instrumental Cirúrgico Ltda). To confirm the specificity of the immunoreactive procedures, adjacent sections were validated according to the above steps, but the primary antiserum was replaced with PBS or normal biotinylated second antibody (instead of primary antiserum). In addition, the specificity of the antisera used in the control sections were incubated with the primary antisera preabsorbed with an excess of its respective antigen in those cases in which the antigen was available. No positive structures or cells were found in these sections. Furthermore, the specificity of the antisera could also be inferred from the localization of the immunostained cells, which agree with previously reports in other neotropical fish species (Nóbrega 2006; Borella et al. 2009). Following immunostaining, the sections were examined and documented using a computerized image analyzer (described above).

**Table 2:** **Primary antisera dilutions used in the immunohistochemical analysis**

Antiserum raised against	Code	Dilution
Chum salmon β-FSH	8510	1:1000
Chum salmon β-LH	8506	1:1000
Chum salmon PRL	8206	1:1000
Chum salmon SL	8906	1:2000
Chum salmon GH	8502	1:1000
Chum salmon β-FSH	2684	1:4000
Chum salmon β-LH	2686	1:4000
Mummichog β-FSH	299	1:1000
Mummichog β-LH	003	1:1000
Mummichog α-GtH	616	1:1000
Carp α-β-GtH	N/S	1:1000
Carp β-GtH	N/S	1:3000

All the antisera used in this study were kindly provided by Drs: H. Kawauchi (School of Fisheries Sciences, Kitasato University, Iwate, Japan), A. Shimizu (National Research Institute of Fisheries Science, Fisheries Research Agency, Kanazawa, Yokohama, Japan) and J. Mancera (Facultad de Ciencias del Mar y Ambientales, Universidad de Cadiz, Spain).

FSH, follicle-stimulating hormone; LH, luteinizing hormone; PRL, prolactin; SL, somatolactin; GH, growth hormone; N/S, not supplied.

### Analysis of data

**Figure 2:**
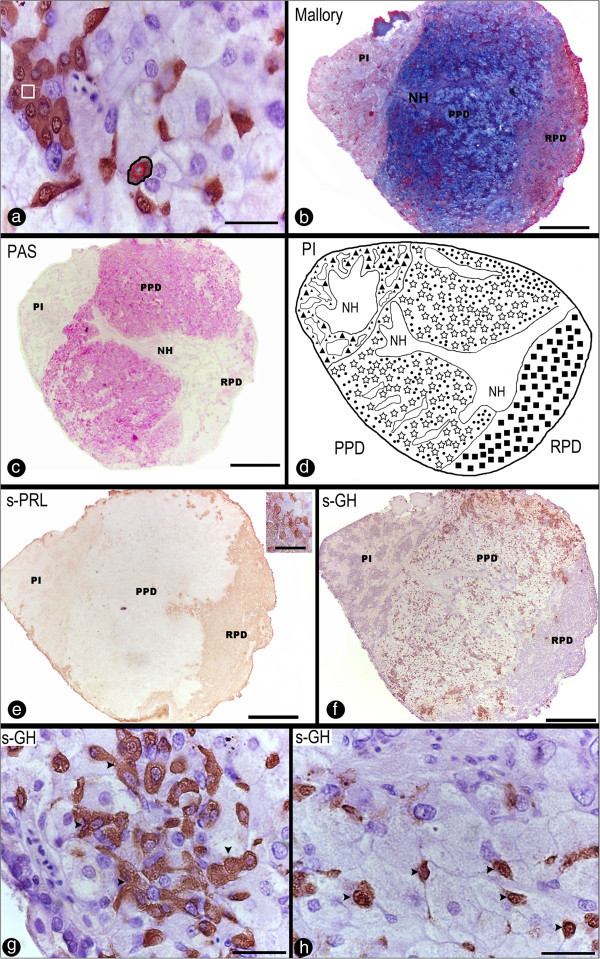
**Sagittal sections through the pituitary gland of*Salminus hilarii*. a)** Photomicrograph of the stained GH cells. Examples of measured nuclear area (red circle) and cell area (black circle) and the optical density (white square) were indicated; **b**-**c)** Sagittal sections of the pituitary stained using Mallory trichrome **(b)** and periodic acid-Schiff (PAS); **d)** Schematic sagittal representation of the pituitary, showing the distribution of ADH cells. RPD: rostral pars distalis; PPD: proximal pars distalis; PI: pars intermedia; NH: neurohypophysis; (■) prolactin cells; (●) growth hormone; (☆) gonadotropins cells; (▲) somatolactin cells; **e)** PRL-ir cells in the RPD. Details of PRL cells using the anti-chum salmon antisera (inset); **f**, **g**, **h)** GH-ir cells in the PPD **(f)** using the anti-chum salmon antisera. Details of cluster (arrowhead) of GH-ir cells **(g)** and isolated (arrowhead) of GH-ir cells **(h)**. Scale bars: **a**, **g**-**h)** 20 μm; **b**-**c**, **e**-**f)** 300 μm (20 μm insert).

For the morphometrical analysis of the pituitary cells during the annual reproductive cycle in both, wild and captive females, the following parameters were used: optical density of the immunostaining, and cellular and nuclear area (Figure 2a).

### Semiquantitative analysis of pituitary cell optical density of staining

For the analysis of optical density of staining, we captured images of each sample using the system LAS (size image: 1260 pixels by 960 pixels). Fifteen cells of each image (ten images per animal) were randomly selected to analyze the optical density of staining (a.u) using Image Gauge version 3.12 software (similar analyses were described in Fiszbein et al. 2010). As it can be difficult to compare staining intensity among tissues processed separately, representatives of females of both conditions and all stages were included in each batch of immunohistochemistry reactions to further control for staining differences.

### Analysis of pituitary cell area and nuclear area

Fifteen cells from each image (ten images per animal) were randomly selected to determine the average cell area. To assess pituitary immunoreactive cell areas (in μm^2^) were measured using Image Pro Plus software (Media Cybernetics). For this analysis, only those cells with detectable nuclei were included in the analysis. In the same fifteen cells, we measured the nuclear area of the pituitary immunoreactive cells (cell-ir) (Image Pro Plus software). These parameters were previously described by Cánepa et al. (2006). To reduce any variability in the histological sections (sections between nucleus and cell), the ratio between these parameters was calculated (nucleus area/cell area).

### Statistical analysis

All values were expressed as mean ± standard error of the mean (M ± SEM). Mean values for each variable were compared taking into account the maturation stage of the animals (previtellogenic, vitellogenic and regression) and the environment in which they were obtained (wild or captive group). Comparisons were made using analysis of variance (one-way ANOVA), followed by the Student-Newman-Keuls (SNK) test for parametric analyses or Dunn’s test (non-parametric analyses). Pearson correlation test comparing environmental and pituitary cells morphometric data were analyzed in both locations throughout the reproductive cycle. In all analyses, differences were considered significant when *P < 0.05*. These analyses were performed using the statistical software SigmaStat for Windows (Version 3.10 Copyright^©^).

## Results

Independent of the environment in which the animal was captured, changes in *S. hilarii* ovary size during gonadal recrudescence were macroscopically observed. During the reproductive cycle, a significant increase in GSI was observed from the previtellogenic to the vitellogenic stage (*P < 0.01* for both groups), and a significant decrease occurred in the regression stage (*P < 0.05* only in wild group (Table 1)). The comparison among females from both groups showed a higher GSI index in the captive group in the regression stage when compared to the wild group (*P < 0.05*) (Table 1).

The correlation analysis between the environmental variables and pituitary cells morphometry showed that there are no significant correlations among them.

### Histological and immunohistochemical analyses of the pituitary

The pituitary gland of *S. hilarii* appeared to be attached to the ventral region of the hypothalamus, to which it was connected by a thin pituitary stalk. The pituitary gland of *S. hilarii* consisted of two components, the NH (neurohypophisis) and the ADH (adenohypophysis) with the last one subdivided in three distinguished areas: the rostral pars distalis (RPD), the anterior part of the gland; the proximal pars distalis (PPD), the central part, and the pars intermedia (PI), the posterior part (Figure 2b, c, d). Additionally, the gland presented distinct cells with different tinctorial properties (Figure 2b, c), and the identification and distribution of the pituitary cells were similar to those described in a wide number of teleost species and will be discussed latter.

The RPD was invaded by thin branches of NH extensions and prolactin cells (PRL) were found in this region. PRL cells were not easily identified by the histochemical methods such as Mallory trichrome, and they were PAS-negative. Thus, we decided to use immunohistochemistry to identify these cells. PRL-ir cells were identified using anti-chum salmon PRL (Figure 2e). This antiserum did not cross-react with other ADH cells; no PRL cells were found outside the RPD region. The PRL cells (cell area 60.87 ± 5.73 μm and 57.66 ± 4.57 μm, in the wild and captive groups, respectively) were weakly acidophilic, and organized in a cordonal arrangement.

**Figure 3:**
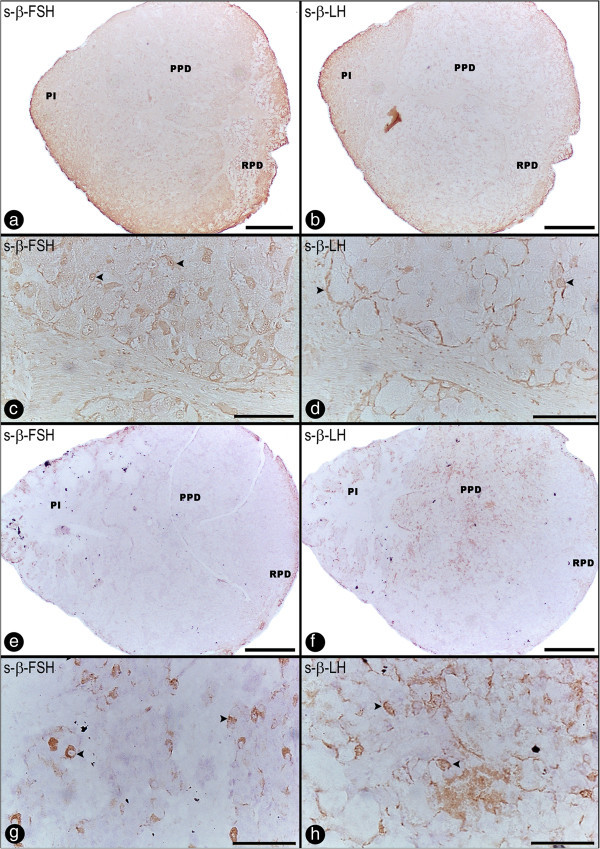
**Sagittal sections through the pituitary gland of*Salminus hilarii*. a**-**d)** β-FSH and β-LH cells using the anti-chum salmon antiserum (Dr. H. Kawauchi). β-FSH-ir cells **(a)** and β-LH-ir cells **(b)** in the PPD showing the weakly immunoreactivity to the anti-salmon. Details of β-FSH cells immunoreactive **(c)** (arrowhead) corresponding to a high magnification of **(a)**. Details of β-LH cells **(d)** (arrowhead) corresponding to a high magnification of **(b)**; **e**-**h)** β-FSH and β-LH cells using the anti-chum salmon antiserum (Dr. A. Shimizu). β-FSH-ir cells **(e)** and β-LH-ir cells **(f)** in the PPD showing the weakly immunoreactivity to the anti-salmon. Details of β-FSH cells **(g)** (arrowhead) and β-LH cells **(h)** (arrowhead) corresponding to a high magnification of **(e)** and **(f)** respectively. For more information, see the explanation in the text. RPD: rostral pars distalis; PPD: proximal pars distalis; PI: pars intermedia. Scale bars: **a**, **b**, **e**, **f)** 300 μm; **c**, **d**, **g**, **h)** 50 μm.

In the PPD region, the NH showed ramified branches close to the cells, and three different cellular types were recognized. The PPD was characterized by the presence of strongly immunoreactive for growth hormone (GH) cells (Figure 2f, g, h), and two weakly immunoreacting gonadotropins cells (β-follicle-stimulating hormone, β-FSH; and β-luteinizing hormone, β-LH, Figure 3a-h). GH cells were immunoreactive with anti-chum salmon (Figure 2f, g, h), which did not cross-react with other ADH cells. GH-ir cells were found throughout the entire PPD region (clusters of GH cells and isolated GH cells were detected in PPD, as depicted in Figure 2g and 2h, respectively), and they were not found in other locations. These cells were PAS-negative, acidophilic cells, showed an oval shape (65.35 ± 4.99 μm and 69.48 ± 2.06 μm, in the wild and captive groups, respectively), contained an eccentric large, irregular and oval nucleus, and were identified next to the NH processes and blood vessels. The gonadotropes were the most abundant and therefore most easily located cells in the PPD, with some cells also found in the PI. These cells were widely distributed in this region (PPD), and histochemical methods revealed that these cells were PAS-positive, basophilic, and contained vacuoles.

We used nine different antisera to identify the gonadotropin cells (GtHs) (three for β-FSH, three for β-LH, and three for general GtHs) by immunocytochemistry. Two types of antibodies (anti-chum salmon, Figure 3a-h) for β-FSH/β-LH (provided by Dr. Kawauchi and Dr. Shimizu, Figure 3a-d and 3e-h, respectively) were used; GtHs cells were weakly immunoreactive to the anti-chum salmon gonadotropins, and the GtHs-ir cells reacted more weakly to anti-chum salmon than to other anti-chum salmon antisera (i.e., anti-chum salmon GH, PRL and somatolactin). Furthermore, the anti-chum salmon β-LH (Figure 3d, h) showed a stronger immunoreaction than β-FSH (Figure 3c, g), in both antibodies.

**Figure 4:**
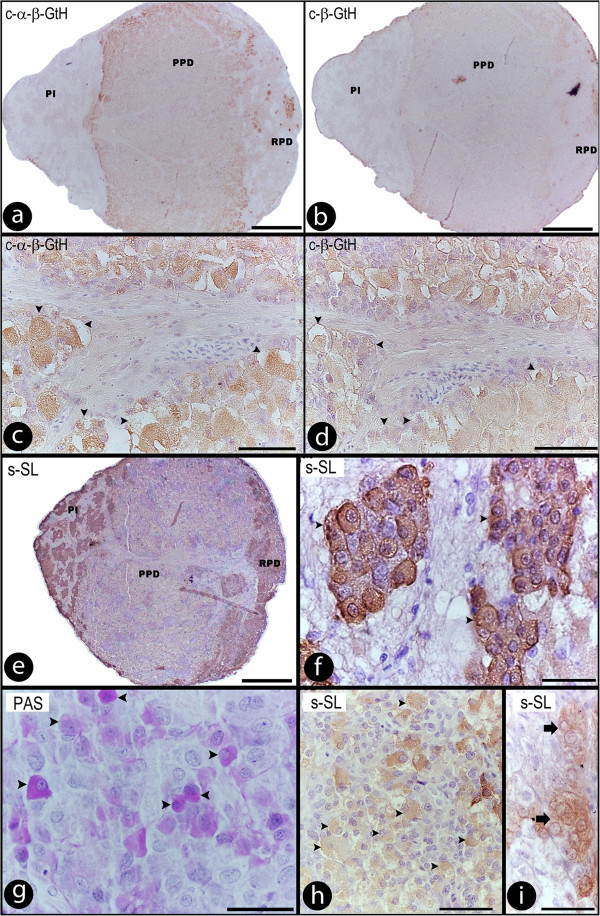
**Sagittal sections through the pituitary gland of*Salminus hilarii*. a**-**d)** Sections of pituitary showing the localization of α-β-GtHs and β-GtHs cells, using single immunocytochemistry, with the anti-carp antiserum (Dr. J. Mancera). **a**, **b)** Gonadotropins cells widely distributed in PPD; **c**, **d)** Details and differentiated GtH-ir cells (arrowhead) using anti-carp antisera, in a major magnitude of **(a)** and **(b)** respectively; **e**,**f)** Sections of the pituitary showing the localization of SL cells using single immunocytochemistry. Details of SL cells (arrowhead) **(f)** in high magnitfication of **(e)**; **g)** periodic acid-Schiff (arrowheads indicate the PAS reactivity to SL cells); **h)** cross-reaction using SL antibody in GtH cells (arrowheads) in PPD region; **i)** PRL cells in RPD region with weakly cross-reaction, when using SL antibody. For more information, see the explanation in the text. RPD: rostral pars distalis; PPD: proximal pars distalis; PI: pars intermedia. Scale bars: **a**,**b**, **e)** 300 μm; **c**,**d**, **i)** 50 μm; **f**, **g**, **h)** 20 μm.

We also identified cells showing immunoreactivity to anti-carp α-β-GtH and anti-carp β-GtH (Figure 4a-d), and these cells had a greater reaction when compared with the immunoreactivity to anti-salmon gonadotropin (Figure 3a-h). Additionally, we also used the anti-mummichog antisera (β-FSH, β-LH and α-β-GtH), though, GtH-ir cells could not be recognized using these antibodies (data not shown).

The PI region was characterized by its large, numerous NH branches and the presence of PAS-positive cells. Somatolactin (SL) cells that were strongly reactive with anti-chum salmon antibody were observed (Figure 4e, f), and no SL cells were found outside the PI. The cells (cell area 69.41 ± 2.53 μm and 89.90 ± 4.93 μm, in the wild and captive groups, respectively) were weakly acidophilic, and PAS-positive (Figure 4g). They showed an oval or elongated shape with an evident nucleus (Figure 4f), and were distributed throughout the entire PI region, next to the NH processes. Anti-chum salmon SL antibody surprisingly immunostained the GtH cells (Figure 4h) in the PPD region (as a result of cross-reaction of the antisera against chum salmon SL with gonadotropins cells in PPD region). On the other hand, SL belongs to the growth hormone family, no cross-reaction between the SL antibody with GH cells were observed, only with weakly cross-reaction with PRL cells (Figure 4i) in the RPD region. In the same way, SL-positive cells were not immunostained with the GH or PRL antibodies.

### Analysis of pituitary cells optical density of staining, cellular area and nuclear area

The weak immunostaining of gonadotropes (compared with the other immunostained, i.e., PRL, SL and GH cells), added to the fact that it was difficult to distinguish if β-FSH and β-LH immunostained different or the same cell types, and did not permit semiquantitative analyses of GtHs.

**Figure 5:**
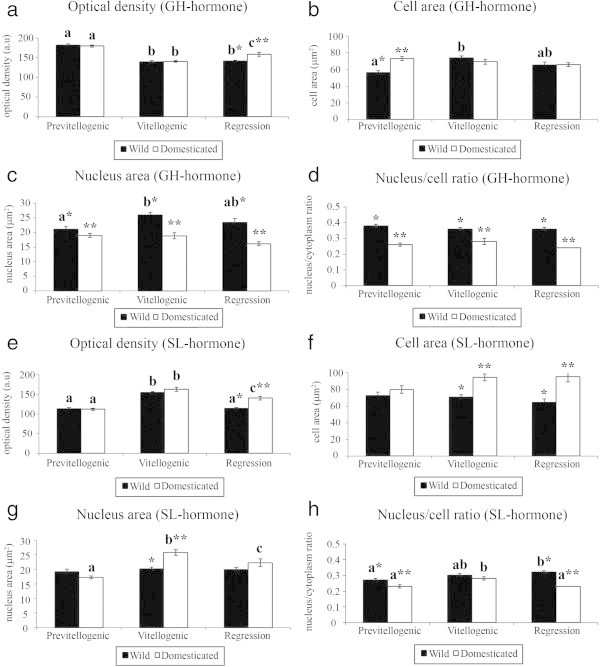
***Salminus hilarii.*Semiquantitative analysis of growth hormone (GH) and somatolactin hormone (SL) in the different environments and ovarian maturation stages. a)** optical density of GH; **b)** cell area of GH; **c)** nucleus area of GH; **d)** nucleus/cell ratio of GH; **e)** optical density of SL; **f)** cell area of SL; **g)** nucleus area of SL; **h)** nucleus/cell ratio of SL. Values followed by different letters **(a**, **b)** are significantly different during the reproductive cycle. Values followed by different symbols (*) are significantly different between the environments. Each bar represents the mean ± SEM. Significance (*P < 0.05*).

The optical density of GH during the reproductive cycle (semiquantitative analyses, Figure 5a) was higher in both groups in the previtellogenic phase, decreased in the vitellogenic stage (*P < 0.05*), and at the regression stage remained constant in the wild females and increased in captive ones (*P < 0.01*). On the other hand, the cell and nucleus area values increased from the previtellogenic to the vitellogenic stages in the wild group (*P < 0.01*), and remained constant in this group at regression stage; but showed no changes in the captive animals (Figure 5b, c). Additionally, between the groups, the nucleus area and nucleus/cell ratio was higher in the wild than in the captive females at all stages (Figure 5c, d; *P < 0.01*).

Semiquantitative analyses of SL showed significant differences during the reproductive cycle and between environments. The optical density showed similar patterns in females from both groups during the reproductive cycle, it increased from the previtellogenic to the vitellogenic stage and then decreased in the regression stage (Figure 5e) (*P < 0.01*) to the values previously found in the previtellogenic phase in the wild group. Despite decreasing in the regression stage, the optical density remained lower in females from the wild than in captive females (*P < 0.01*)*.* We also observed an increase in the cell and nucleus area with the onset of vitellogenesis in the captive group (Figure 5f, g). At this stage, between the groups, both the cell and the nucleus areas were larger in captive animals than in wild females (Figure 5f, g) (*P < 0.01*) and the nucleus area then decreased during the regression phase, but only in captive females (*P < 0.01*). The same patterns of variation were found in the nucleus/cell ratio (Figure 5h) in captive females. Furthermore, the nucleus/cell ratio was higher in wild than in captive females during the previtellogenic and regression stages (*P < 0.01*).

Semiquantitative analysis of PRL showed no significant differences neither throughout the reproductive cycle, in both groups, nor between wild and captive females.

## Discussion

Previous studies on the reproductive cycle of *S. hilarii* showed that animals captured in different points of Tietê River did not differ in terms of morphological or anatomical arrangement of ovaries (Honji et al. 2009), therefore, we assumed that they belong to the same group. Additionally, as expected, GSI values increased independently of the environment, wild or captivity (Honji et al. 2009). In this way, as the ovaries grow in *S. hilarii*, the largest GSI values were found in animals with fully developed ovaries (vitellogenic stage), and the lowest GSI values were registered during the previtellogenic stage, suggesting a normal ovarian development even in captivity. On the other hand, captive females showed a dysfunction in the endocrine system when compared to wild animals. It seems that the maintenance in captivity blocks spontaneous ovulation and consequently reproduction, demonstrating the importance of basic studies of the pituitary-gonad axis, as suggested by Amaral et al. (2007) and Honji et al. (2009,2011), for *S. hilarii* females.

Although the ADH and the distribution of adenohypophyseal cells in *S. hilarii* were similar to those described in a large number of teleost species (for reviews, Kawauchi and Sower 2006; Levavi-Sivan et al. 2010), the identification and detailed distribution of the various ADH cell types in reophilic fish, have not been performed yet in South American teleost species. Beyond the identification of these cells, the comparison of them between wild vs. captive females throughout the reproductive annual cycle and different environments have never been performed in potamodromous fish.

GtHs play critical roles in the regulation of reproductive processes, optimal rates of synthesis and secretion of GtHs are necessary and critical for successful gonadal maturation and spawning (Zohar et al. 2010; Levavi-Sivan et al. 2010). In captivity, neotropical females tend to exhibit reproductive dysfunctions, which include the failure to undergo final oocyte maturation and an absence of egg production or reduction in egg quality and quantity (Zohar et al. 2010; Bobe and Labbé^2010^), as observed in *S. hilarii* (Honji et al. 2009,2011). Furthermore, failure to spawn in captive females has been identified to be due to dysfunctions in the release of GtHs from the pituitary gland (Guzmán et al. 2009). Therefore, the endocrine system of females is adversely affected, and as the normal functioning of GtHs release is changed, the reproductive system fails (Mylonas et al. 2010; Levavi-Sivan et al. 2010). However, before planning the manipulation of ADH hormone secretion, which will enhance fish reproduction in captivity, it is first necessary to establish the morphometrical parameters and cell physiology of all cell types involved in the reproductive process.

To determine if β-FSH and β-LH are produced by only one type of cell or by different cell types in the *S. hilarii* pituitary gland, two adjacent sections of the pituitary were immunostained with β-FSH and β-LH antibodies and the staining was compared at high magnification. Nevertheless, it was difficult to distinguish if β-FSH and β-LH immunostained different cells or the same cell type, because different adjacent sections generally showed different patterns of immunostaining in many cells in the PPD region; additionally, in others cells, there were also cells immunostained with both antibodies.

In the present study, we showed that pituitary gonadotropes are more immunoreactive to β-LH antibodies than to β-FSH antibodies. This result can be associated to the fact that the molecular structure is more conserved, both in structure and primary sequence, in fish β-LH subunit than in β-FSH (Levavi-Sivan et al. 2010). Further research with the aim to investigate this question, using specific antisera for Characidae family, will be necessary to answer this question regarding gonadotropins in South America fishes, including *S. hilarii*. Additionally, due to lack of strong positive immunoreactions with GtHs cells in *S. hilarii*, our group decided to study in parallel with the present study, the β-FSH and β-LH gene expression in wild and captive females (Moreira et al. in preparation).

GH, SL, and PRL constitute a family of structurally related hormones (Kawauchi and Sower 2006) that have multiple physiological functions. A number of investigations on the GH family in fish were focused on their pituitary distribution and the characterization of these hormones. However, few studies have been conducted on seasonal variations in these molecules or the influence of environmental changes on the GH family in non-salmonid fish species (Vargas-Chacoff et al. 2009; Fiszbein et al. 2010), and no studies on the GH family in South America fish (reophilic Neotropical fish species), have assessed the impact of a migration impediment on these cells. In this study, we used anti-chum salmon GH, which showed a quite specific immunostaining within the GH cells of *S. hilarii*, as demonstrated similarly in other teleost fishes. The distribution of GH cells followed the patterns already described in others fish species (Vissio et al. 1997; Segura-Noguera et al. 2000; Pandolfi et al. 2001a; Sánchez-Cala et al. 2003; Borella et al. 2009). GH-ir cells have been located in the PPD region, either isolated or clustered, and no cross-reactivity of other cell types occurred in *S. hilarii*. However, some species showed cross-reactivity of GH antisera with PRL cells and of PRL antisera with GH cells or with PAS-positive cells of the PI (SL cells) (García-Hernández et al. 1996; García-Ayala et al. 1997).

Our results clearly demonstrate that GH is potential involved in the *S. hilarii* reproductive cycle. GH cells in vitellogenic females were significantly larger cells and had larger nucleus areas than did GH cells in previtellogenic females in the wild. Additionally, females from both environments showed a lower optical density from the previtellogenic to vitellogenic stages. This result could be correlated with major cellular synthesis activity, and probably with an increase in hormone release. In *Sparus aurata*, the pattern of GH protein expression showed differences during the seasons, with the highest values recorded during the summer (reproductive period) and the lowest values recorded during autumn (Vargas-Chacoff et al. 2009). In the same species, plasma GH levels increased progressively during late spring-early summer, whereas during autumn/winter, circulating GH remained low (Mingarro et al. 2002). Considering that *S. hilarii* vitellogenic females were captured mainly during spring/summer and the previtellogenic/regression animals were caught mainly in the autumn/winter, our results were consistent with the variation of GH profile reported during the reproductive cycle. Additionally, such increases in GH cell activity in the vitellogenic stage could be related to increased feeding during this period, as was observed in other teleost fish (Holloway and Leatherland 1998). It is also probable that in this species, GH secretion is regulated by melatonin, as observed in rainbow trout (*Oncorhynchus mykiss*), and/or by GnRH as suggested by Canosa et al. (2008), who demonstrated that the circulating levels of LH and GH increase at the time of ovulation in goldfish.

In this study, SL cells in *S. hilarii* were small, weakly acidophilic, and PAS-positive. They showed an ovoid or elongated shape with a conspicuous nucleus and were distributed in all regions of the PI, close to the NH processes. Whereas the SL cells were PAS-positive in *S. hilarii*, PAS methods in several other teleosts have revealed two different types of SL-ir cells in the PI region. Biochemical analyses have demonstrated both the existence and the absence of N-glycosylation sites in the SL. Briefly, in salmonid fish, SL-ir cells were PAS-negative as a result of the non-glycosylated SL form (Rand-Weaver et al. 1991; Kaneko 1996). PAS-negative (non-glycosylated form) SL-ir cells were also observed in some other non-salmonids such as *S. aurata* (Villaplana et al. 1997), *Diplodus sargus* (Segura-Noguera et al. 2000), *Trachinus draco* (Sánchez-Cala et al. 2003) and *Arapaima gigas* (Borella et al. 2009). However, PAS-positive cells, as a result of the glycosylated SL form, were identified in several fishes (Rand-Weaver et al. 1991; Kaneko 1996). In addition, SL hormone in *S. aurata,* was observed as both the glycosylated and non-glycosylated form in adults (Cavari et al. 1995). These data are similar to those reported for *Solea senegalensis,* which showed PAS-positive and PAS-negative cells in the same species (Pendón et al. 1998). Different forms of SL hormones were also observed during the ontogeny of *Cichlasoma dimerus*, with the glycosylated form of SL being expressed in adults, and a non-glycosylated form of SL appearing in larvae (Pandolfi et al. 2001b). In *S. hilarii*, SL it is probably glycosylated, considering that SL-ir cells were PAS-positive in this species.

The distribution and localization of SL cells in *S. hilarii* is similar to other teleosts using antisera against chum salmon SL (see review of Kaneko 1996; Kawauchi and Sower 2006). However, some species showed reactions to anti-GH and anti-PRL in the PI. These results could possibly be due to a cross-reaction of these antisera (GH and PRL) with SL cells because of structural similarities. Although the migration of cells (GH and PRL) to the PI region during the pituitary development cannot be excluded (Farbridge and Leatherland 1986), our results showed a cross-reaction of antisera against chum salmon SL with PRL cells in the RPD region and GtHs cells in PPD region. Our pre-adsorption tests and histology method (PAS stained) confirmed this cross-reaction among the SL antisera with PRL and GtHs cells. Similar cross-reactions were reported with anti-gonadotropin in SL cells in *Xiphophorus maculatus* and *Poecilia latipinna* (Margolis-Kazan et al. 1981; Batten et al. 1975; Batten 1986). According to these authors, the SL and GtHs hormone can contain antigenically similarity, which might be difficult to remove during biochemically purification.

The larger nuclear area and more intense optical density in SL cells in pituitary of vitellogenic *S. hilarii* suggest a possible role of SL in the reproductive physiology of this species. Taking into consideration the variation of SL hormone during the reproductive cycle, Mousa and Mousa (1999,2000) identified seasonal variations in the synthesis and secretion of SL during sexual maturation and spawning in *Oreochromis niloticus* and *Mugil cephalus*. In *O. niloticus* the synthetic and secretory activity of the SL-ir cells increased during sexual maturation and spawning (Mousa and Mousa 1999). In coho salmon, SL levels increased during smoltification and decreased when the smoltification was complete. They then remained low until prespawning, reaching a peak at spawning (Rand-Weaver et al. 1992). During the vitellogenesis process, there is an increasing demand for calcium due to the involvement of this cation in the transport of vitellogenin from the liver to support the development of the embryo (Lubzens et al. 2010). This suggests that SL is indirectly or directly linked with reproductive physiology (Kakizawa et al. 1993,1995). Our results regarding the cellular morphometrical parameters of SL in captive animals are consistent with studies in *M. cephalus*, in which the synthetic activity of SL was higher in captivity than wild animals (Mousa and Mousa 2000). Other studies suggest that SL may be responsible for the regulation of associated processes that indirectly affect reproductive activity (Vissio et al. 2002), i.e., metabolism (especially, with lipogenic enzymes and lipid mobilization), temperature variation (Mingarro et al. 2002; Vargas-Chacoff et al. 2009), feeding, and ionoregulation (Kakizawa et al. 1995).

The present results also show that chum salmon PRL antiserum can be recommended for the identification of PRL cells in *S. hilarii*. The distribution of these cells followed the patterns already described in other teleost fishes (Kawauchi and Sower 2006). PRL-ir cells have been located in the RPD region, and no cross-reactions with other cell types occurred in this species. However, some other species showed cross-reaction of PRL antisera with GH cells (Pandolfi et al. 2001a). Additionally, our results indicated no changes in PRL pituitary content during sexual maturation and also no differences between the groups (wild and captive females). Several studies have analyzed the pituitary PRL in teleost species, and it was established that PRL is an important hormone involved in osmoregulatory processes in these animals. It is essential for ion uptake as well as for reducing ion and water permeability of osmoregulatory surfaces (Sakamoto and McCormick 2006). Furthermore, when important differences in temperature and photoperiod occur, variations in PRL gene and hormone expression levels were observed in fish, suggesting that the seasonal cycle of PRL is also influenced by seasonal acclimatization (Figueroa et al. 1997; Vargas-Chacoff et al. 2009). In *C. dimerus*, pituitary levels of PRL were significant higher when animals were exposed to a long photoperiod rather than a short photoperiod (Fiszbein et al. 2010). Further, in *Cyprinus carpio*, it has been shown that photoperiod constitutes a particularly relevant modulator in the neuroendocrine cascade that activates PRL transcription (Figueroa et al. 1997), due to the modulation of PRL secretion by melatonin, a highly conserved feature in vertebrates (Falcón et al. 2007). In *S. hilarii*, even considering the annual variations of water temperature and photoperiod, there was no difference in PRL content, as described in the other fish species.

## Conclusions

In conclusion, this study in *S. hilarii* broadly supports the idea that beyond the gonadotropes, other adenohypophyseal hormones are involved in reproduction in teleost fish and may be responsible for the regulation of associated processes that indirectly affect reproductive status. Our data, together with the findings previously described by Amaral et al. (2007), who observed dysfunction in GtHs and in progestagen synthesis, in captive females, suggest that the reasons for the reproduction failure of migratory fish in captivity are complex. Thus, analyses of the brain, especially the higher control of the hypothalamic gonadotropin-releasing hormone (GnRH), should also be conducted.
